# Immunoinformatics Aided Design and *In-Vivo* Validation of a Cross-Reactive Peptide Based Multi-Epitope Vaccine Targeting Multiple Serotypes of Dengue Virus

**DOI:** 10.3389/fimmu.2022.865180

**Published:** 2022-06-21

**Authors:** Vikas Kaushik, Sunil Krishnan G, Lovi Raj Gupta, Utkarsh Kalra, Abdul Rajjak Shaikh, Luigi Cavallo, Mohit Chawla

**Affiliations:** ^1^ Domain of Bioinformatics, School of Bio-Engineering and Bio-Sciences, Lovely Professional University, Punjab, India; ^2^ Department of Research and Innovation, STEMskills Research and Education Lab Private Limited, Faridabad, India; ^3^ Department of Data Science, Innopolis University, Innopolis, Russia; ^4^ Kaust Catalysis Center, Physical Sciences and Engineering Division, King Abdullah University of Science and Technology (KAUST), Thuwal, Saudi Arabia

**Keywords:** immunoinformatic analysis, dengue (DENV), molecular docking & molecular dynamics (MD) simulation, vaccine design for emerging infections, multi epitope peptide vaccine, *in vivo* study

## Abstract

*Dengue virus* (DENV) is an arboviral disease affecting more than 400 million people annually. Only a single vaccine formulation is available commercially and many others are still under clinical trials. Despite all the efforts in vaccine designing, the improvement in vaccine formulation against DENV is very much needed. In this study, we used a roboust immunoinformatics approach, targeting all the four serotypes of DENV to design a multi-epitope vaccine. A total of 13501 MHC II binding CD4+ epitope peptides were predicted from polyprotein sequences of four dengue virus serotypes. Among them, ten conserved epitope peptides that were interferon-inducing were selected and found to be conserved among all the four dengue serotypes. The vaccine was formulated using antigenic, non-toxic and conserved multi epitopes discovered in the in-silico study. Further, the molecular docking and molecular dynamics predicted stable interactions between predicted vaccine and immune receptor, TLR-5. Finally, one of the mapped epitope peptides was synthesized for the validation of antigenicity and antibody production ability where the *in-vivo* tests on rabbit model was conducted. Our *in-vivo* analysis clearly indicate that the imunogen designed in this study could stimulate the production of antibodies which further suggest that the vaccine designed possesses good immunogenicity.

## Introduction

Dengue is one of the most frequent arboviral diseases and presents a major health concern for people living in tropical and sub-tropical climate regions of the world and is present in over 125 countries (1). Mosquitoes of the genus *Aedes* are principally responsible for transmitting dengue virus (DENV) to human beings which is affecting 400 million people worldwide annually (2).

The DENV genome is composed of nearly ~ 11 kb of single and positive sense RNA, which encodes for a protein comprising 3391 amino acids (3, 4). DENV possesses four closely related serotypes (DENV 1-4) (5). However, a fifth serotype has also been reported recently (6). Remarkably four closely related dengue serotypes viz. DENV-1, DENV-2, DENV-3, and DENV-4 share 65-70% genome similarity (7).

Dengue disease ranges from mild self-limiting dengue fever (DF) to severe dengue hemorrhagic fever (DHF) and dengue shock syndrome (DSS). Unfortunately no drugs, vaccines or targeted therapies specifically for controlling dengue virus have been available till date (8). However proper intensive care is the only choice available in order to treat dengue. It is well known that vaccines are an effective way to control infectious diseases and also provide immunity against pathogens. The first and only commercialized dengue vaccine available till date is Dengvaxia (CYD-TDV) (9). Dengvaxia vaccine hesitancy started due to secondary heterotypic infection, long-lasting cross-protection, side effects and safety related issues (9, 10). Besides Dengvaxia, many other vaccine formulations against DENV are still under clinical trials (11) that includes TAK-003, which is a live attenuated tetravalent vaccine, containing both structural and non-structural proteins, and which is currently under phase III clinical trial and with a reported efficacy of 80% (12). TAK-003 which was shown to be effective against all four DENV serotypes, albeit producing low levels of antibodies for DENV3 serotype (13). Next potential vaccine includes a tetravalent DNA vaccine, which is also in phase III clinical trials (14). Another vaccine formulation, V180 that is a genetic construct of a tetravalent vaccine formulation, based on DENV envelope glycoprotein is under phase I clinical trials (11).

Despite all the current developments in vaccine design, improvement in vaccine design and formulation is essential, especially due to the tetravalent nature of DENV (11). Secondary infection with a different DENV serotype from primary infection could be even more lethal (15). Therefore, a serotype-specific cross-protection is an utmost requirement for an effective immunity (16, 17).

With the recent advancement in the field of bioinformatics, different strategies have been employed to design knowledge based vaccines using the immunoinformatics approach. Using an immunoinformatics approach, multi-epitope vaccines have been designed against various pathogens including Epstein–Barr virus and Chlamydia trachomatis virus species (18–20) that show a promising cellular and humoral response and have further been shown to work both *in vitro* and *in vivo* murine model.

Further, a plethora of immunoinformatics based studies are available in literature in the context of designing DENV vaccine where various research groups have been working on identification of epitopes from various structural and non-structural proteins from DENV virus (21–24). In particular, identification of highly conserved amino acid sequences for the entire DENV proteome was performed by Khan and coworkers (25) where they identified highly conserved and immune relevant DENV sequences that were common across the four serotypes and have direct implications in design of tetravalent vaccine (26). A very similar study by same group focused on identification of highly conserved and serotype specific DENV peptides that are potentially immune-relevant which may be relevant candidates for vaccine design as such sequences minimize the issue of altered peptide ligands (APLs) that are cross-reactive between the dengue serotypes (27). In one of our previous studies, we have also applied immunoinformatics approach and discovered the CD8+ epitope peptides namely TSEIQLTDY, IGIGILLTW and IAVGMVTLY from dengue virus envelope, specifically from DENV-1 serotype (28). Further, in one of our recent studies, epitope peptides specifically HTLWSNGVL and FTTNIWLKL from dengue non-structural, NS1 protein that were able to form a stable complex with MHC I HLA allele (29).

In the present work we have utilized immunoinformatics approach in order to design a candidate multi-epitope vaccine that could work against all the four serotypes of DENV 1-4 and is likely to be immunogenic. Recent experimental studies have clearly indicated the the role of dengue-specific CD4+ T cells to control dengue infection *in vivo*, which could be the potential target for dengue virus vaccine development (30, 31). Therefore, we have particularly focused on the prediction of CD4+ T-cell epitope peptides which follow two pathways to produce both T cell immunity and B cell immunity and altogether cell mediated and humoral immunity would be invoked. Suitable adjuvants and linkers were added during the construction of multi-epitope vaccine construct. The physiochemical properties along with structuiral and antigenicity properties were carefully evaluated for predicted multi-epitope vaccine construct, along with its interaction with immune receptor, TLR-5. Finally, one of the potential epitopes that was conserved among all the four DENV serotyoes was synthesized and has been tested *in-vivo* on a rabbit model.

## Methodology

### Protein Sequences Identification and Antigenicity Analysis

UniProtKB/Swiss-Prot database from https://www.uniprot.org/uniprot/ provides manually annotated, accurate, and reviewed protein data. The genome polyprotein sequences of DENV serotypes DENV-1, DENV-2, DENV-3, and DENV-4 having the UniProtKB Id: P33478, P07564, Q6YMS4, and Q58HT7 respectively were selected and retrieved from the UniProtKB/Swiss-Prot protein database. The antigenicity prediction server VaxiJen 2.0 (32) was used for the assessment of the antigenic protein sequence. This tool was based on auto cross-covariance (ACC) transformation on virus protein sequence dataset derived models predicted the antigenicity of the protein sequence (33).

### MHC II Binding T Cell Epitope Prediction

The MHC II molecules have an important role in T cell antigen presentation and immunity. An artificial neural network-based NetMHCIIpan version 4.0 had been reported as a powerful tool to predict MHC II binding CD4+ T cell epitope peptides (34). This prediction tool was used parameters like Binding Affinity (BA) and Eluted Ligand mass spectrometry (EL) scores to find the epitope peptide at any length.

### Interferon Inducing Epitope Prediction From CD4+Epitopes

To design the most effective subunit vaccine candidate, identification of epitopes with the ability of interferon-gamma induction from MHC-II binding epitopes was done using the IFN epitope server (35).

### Mapping and Analysis of CD4+ T Cell Epitopes

The predicted MHC II binding CD4+ T cell epitopes were screened and analyzed for the identification of potential epitopes for efficacious vaccine design. The CD4+ T cell epitopes were mapped and analyzed by various epitope analysis tools. The conservancy of epitope peptides was analyzed by Immune Epitope Data Base (IEDB) analysis resource tool (36). This tool facilitates the identification of the linear degree of the conservancy of an epitope and epitope-based vaccine design (36). The peptide toxicity was predicted by the ToxinPred server (37). This tool predicted nontoxic epitopes from the antigenic epitope sequence dataset (37).

### Physico-Chemical Properties of T Cell Epitopes

The predicted epitope was analyzed for physical/chemical properties using the ProtParam (38) algorithm of Expasy web server https://web.expasy.org/protparam/. The raw sequence of the epitope is used to compute the molecular weight, theoretical pI, amino acid composition, atomic composition, extinction coefficient, estimated half-life, instability index, aliphatic index, and grand average of hydropathicity (GRAVY) score. The calculated properties have been reported in **Table S1**, see **Supplementary File**.

### Multi Epitope Vaccine Formulation

For the design and formulation of a multi-epitope vaccine, three interferon-inducing CD4^+^ T cell epitope peptides were used (see Results and Discussion section). The epitope peptides were selected based on immunoinformatics epitope mapping. The protocol adopted in the current study is in line with the previous studies where an *in-silico* peptide vaccine formulation approach was already employed to design vaccines for Human Papilloma Virus (39), Herpes Simplex Virus type 1 and 2 (40) Dengue virus (29).The multi-epitope vaccine construct contains three interferon-inducing CD4^+^ T cell epitopes, three adjuvants, and a linker. The VaxiJen server (32) was used to compute antigenicity and protparam (38) was used to compute physical and chemical parameters of epitope peptides, see **Table S1** and multi-epitope vaccine. Finally the solubility was predicted using SCRATCH protein predictor (41).

### Molecular Modeling, Docking and Molecular Dynamic Simulations of Designed Multi-Epitope Vaccine With TLR-5 Receptor

To predict the 3D structure of multi-epitope vaccine and Human TLR-5 receptor molecule we used Alphafoldv2.0 program (42, 43). For docking of multi-epitope vaccine and TLR-5 receptor complex, the HADDOCK server (44) was used. Further, all the Molecular dynamic (MD) simulations were performed with the GROMACS 2019 simulation program (45). TLR5-vaccine was placed in a cubic box and solvated with TIP3P water molecules creating a solvent layer at least 12 Å thick. The Amber ff99SB-ILDN (46) force field was used to model the parameters of proteins. Charge was neutralized adding appropriate number of K^+^ ions and extra K^+^Cl^-^ ions were added to achieve a bulk ionic strength of 0.15 M using the Joung-Cheatham ion model (47). The simulation box contains 224932 water molecules, 607 K^+^ ions and 601 CL^-^ ions. The total number of atoms in the system was 690620. The system was first minimized with 50000 steps of steepest descent method with 1000 kJ/mol nm^2^ position restraint on protein heavy atoms. Further, minimization was carried out without any restraint on protein. Equilibration of each system was carried out in a phased manner. First, 100 ps NVT simulation was carried out with restraint on heavy atoms of the protein. It was followed by 100 ps NPT simulation with restraint on heavy atoms of the protein. Production simulations were performed using the NPT ensemble for 100 ns. Three different trials were carried out. The temperature was maintained at 300 K using velocity rescaling with a coupling time of 0.1 ps. The pressure was maintained at 1 atm for NPT simulations using a Parrinello–Rahman barostat (48) with a coupling time of 2 ps. Equations of motion were integrated using the leapfrog algorithm with a time step of 2.0 fs. The total electrostatic interactions were evaluated using the particle mesh Ewald (PME) summation (49). Coulomb and van der Waals cut-offs of 1.0 nm were employed. Periodic boundary conditions in all directions were employed to mimic the bulk behavior. Bond lengths with hydrogen were constrained with the LINCS algorithm (50). Coordinates have been collected in trajectory files every 10 ps. Trajectory processing and most of the analysis have been performed using the GROMACS tools. The visualization and molecular graphics images were created using the PYMOL (51) and VMD software (51). Graphs were plotted using Excel 2016.

### Peptide Synthesis and Conjugation

One of the potential antigenic peptide with CKREKKLGEFGKAKG sequence was synthesized using the GenScript’s microwave-based PepPower™ technology merging Solid-phase peptide synthesis (SPPS) and Liquid-phase peptide synthesis (LPP) and Microwave technology. The peptide concentration was measured by using NanoDrop Spectrophotometer absorbance at 280nm.The purity was measured by SDS-PAGE and antigen with an expected purity of ≥85%.The resulting peptide was added with a cysteine residue in the N –terminal and is conjugated with KLH (Keyhole Limpet Hemocyanin) protein using MBS (m-maleimidobenzoyl-N-hydroxysuccinimide ester) method.

### *In-Vivo* Validation for Antibody Development and Assessment

KLH conjugate peptide was inoculated in New Zealand rabbits (n=2) following the PolyExpress immunization method. This method was used in anti-KRV envelope polyclonal antibody generation (52). The antibodies were retrieved and the purity of the polyclonal antibody was determined performing SDS-PAGE. Further, concentration of the antibody was measured by NanoDrop Spectrophotometer at 280 nm. Afterwards, the antibody titer was determined with Indirect ELISA using IgG as the control. The antigen used was a synthesized peptide with a coating concentration of 4ug/ml, and 100μl/well in ELISA plate. The anti-rabbit horseradish peroxidase(HRP) conjugated IgG was used as the secondary antibody. The OD_450nm_ value was measured using an ELISA plate reader.

## Results and Discussion

### Retrieved Protein Sequence and Analysis

For an efficacious vaccine against DENV, the requirement of potency against all four serotypes (DENV 1-4) should be established. Four sequences corresponding to the four serotypes were selected Dengue virus type 1(DENV-1), a strain fom Singapore/S275/1990; Dengue virus type 2(DENV-2), a strain from Jamaica/1409/1983; Dengue virus type 3 (DENV-3), a strain from Sri Lanka/1266/2000; and finally Dengue virus type 4 (DENV-4), a strain from Philippines/H241/1956 that were isolated from DENV epidemic countries. The VaxiJen identified for four protein sequences were antigenic with an antigenicity score >0.4. The antigenic protein sequence could be a potential vaccine candidate. The immunoinformatics approach adopted here is fast and cheaper than the current available experimental methods used in reverse vaccinology for the discovery of potential antigenic vaccine candidates (33, 53, 54).

### MHC II Binding CD4+T Cell Epitopes

The NetMHCIIpan version 4.0 (34, 55) predicted a total of 13501 CD4+ epitope peptides corresponding to DENV 1-4 serotypes which were predicted to have a good binding with MHC II receptor alleles DRB1*0101, DRB1*0401, DRB1*0701, DRB5*0101, DRB1*1501, DRB1*0901, and DRB1*1302. The seven clusters of HLA-DR alleles of MHC class II were selected due to these alleles being found in 95% of the human population. A default threshold value of 2% and 10% rank was set for the prediction of strong and weak MHC II binding peptides respectively (34, 55). Of 13501 peptides, 137 epitopes were selected using binding affinity score. Further, 51 epitopes were screened based on their antigenicity potential. Out of 51 antigenic epitopes, 31 were screened to be non-toxic, and this number was further reduced down to 10 that were IFN-gamma inducing epitopes. Finally, based on the conservancy analysis, selection of three epitope peptides was made where all the three epitope peptides were found to be conserved in all the four DENV serotypes, see below and also supplementary information.

### Interferon Inducing CD4+ T Cell Epitopes

From the 13501 CD4+ epitopes interferon inducing CD4+ epitope peptides were predicted using the IFN epitope server (35). The selected 10 IFN-gamma inducing epitopes are EGKIVGLYGNGVVTT, REGKIVGLYGNGVVT, GKIVGLYGNGVVTTS,TFTMRLLSPVRVPNY,SADLSLEKAAEVSWE, ATFTMRLLSPVRVPN, SSADLSLEKAAEVSW, KREKKLGEFGKAKG, KATYETDVDLGSGTR and VLRGFKKEISNMLN were considered for further analysis.

### Conservancy, Antigenicity, and Toxicity of CD4+ T Cell Epitope Peptides

The usage of conserved epitopes would be an ideal choice in order to design vaccines that would eventually provide broader protection across DENV 1-4 strains. The conservancy analysis was done through IEDB conservancy analysis resource (36). The degree of linear conservancy of 10 interferon-inducing epitope peptides within the given four DENV 1-4 protein sequences was set at 100% identity level. The analysis revealed that the three epitopes were showing 100% of protein sequence matches for DENV 1-3 serotypes. The potential epitopes ‘ATFTMRLLSPVRVPN’ and ‘TFTMRLLSPVRVPNY’ shows 80% sequence identity with DENV-4 sequence. However, a higher sequence identity of 92.86% was noted for ‘KREKKLGEFGKAKG’ epitope with DENV 4 protein sequence. The highly conserved CD4+ epitope peptides KREKKLGEFGKAKG, TFTMRLLSPVRVPNY and ATFTMRLLSPVRVPN were selected and summarized in the **Table 1**. All the selected epitopes were predicted to be non-toxic and antigenic. The physical and chemical properties of the epitope analyzed and the results are summarized in **Table S1**, see **Supplementary File**.

**Table 1:** Selected epitope peptide conservancy analysis.

Epitope Number	Epitope sequence	Percent of protein sequence matches DENV1	Percent of protein sequence matches DENV2	Percent of protein sequence matches DENV3	Percent of protein sequence matches DENV4	Antigenicity	Toxicity
1.	ATFTMRLLSPVRVPN	100	100	100	80	ANTIGEN	Non-Toxin
2.	KREKKLGEFGKAKG	100	100	100	92.86	ANTIGEN	Non-Toxin
3.	TFTMRLLSPVRVPNY	100	100	100	80	ANTIGEN	Non-Toxin

### Dengue Vaccine Formulation and Analysing its Physico-Chemical Properties

**Figure 1:**
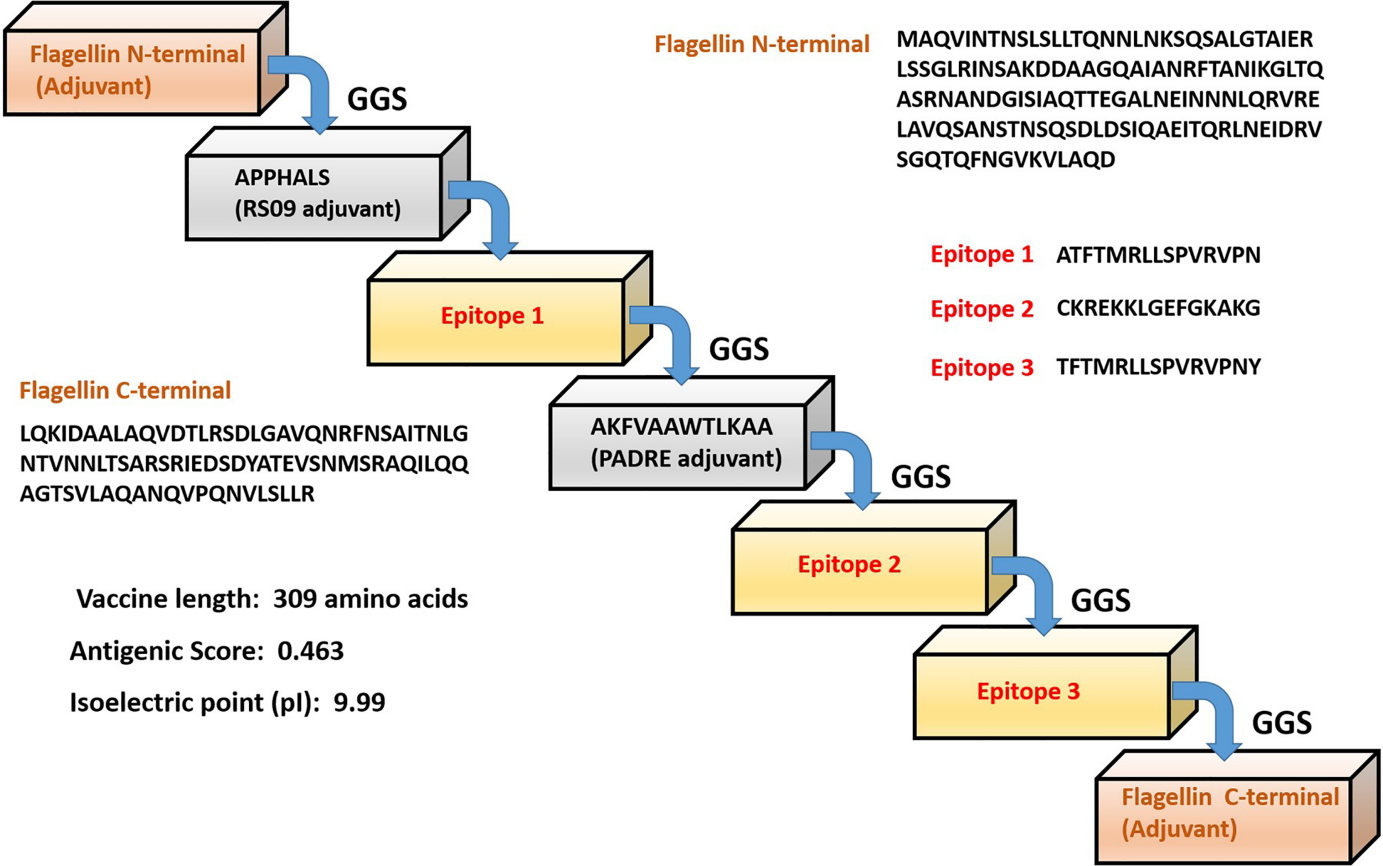
Schematic representation of the predicted multi-epitope vaccine construct with different adjuvants, linkers and epitopes with their respective amino acid sequence.

The formulated polyvalent epitope peptide vaccine consists of 309 amino acid residues. This includes three immunogenic T cell epitopes; three adjuvants (RS09 having sequence APPHALS; PADRE having sequence, AKFVAAWTLKAAA; and an N-terminal and C-terminal sequence of *Salmonella typhimurium* flagellin protein that were linked to the epitopes with the help of GGS linker as an immune-adjuvant to elicit a robust immune response (56, 57). In fact it was shown that the flagellin’s C terminal D0 domain is required for TLR5 receptor activation (56, 57). The sequence of formulated vaccine is shown is **Figure 1**.

The formulated vaccine was antigenic with an antigenic score of 0.463 as calculated by VaxiJen 2.0 server. The final vaccine has molecular weight of 32.6 kDa, and isoelectric point (pI) of 9.99. The total number of negative and positive charged amino acids were 22 and 32 respectively. The calculated aliphatic index (88.19) indicates a stable protein in a broad range of temperatures. Our vaccine presented an estimated half-life of 30 hours in mammalian reticulocytes (*in vitro*), and with a >20 hours (in yeast, *in vivo*) and >10 hours (Escherichia coli, *in vivo*) analyzed by Expasy ProtParam server. Finally the predicted solubility of the vaccine was calculated to be 0.6909 upon overexpression in *E.coli*.

### Modeling and Docking of TLR-5 and Multi-Epitope Vaccine Construct

**Figure 2:**
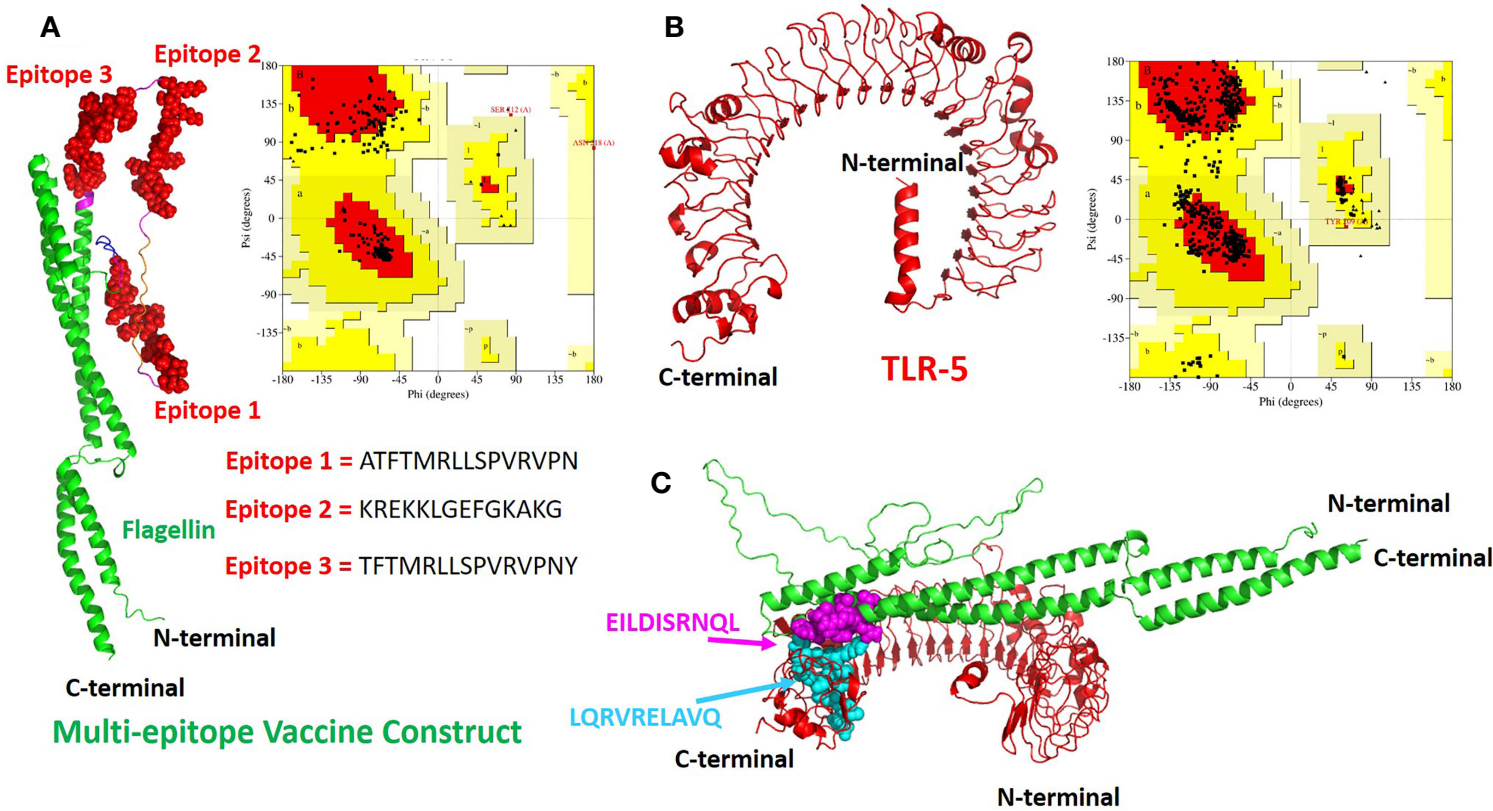
The modeled 3D structures of the **(A)** multi-epitope vaccine construct. The three epitopes inserted are shown in red spheres and the bacterial flagellin sequence is present at the N- and C-terminal of the vaccine construct and is colored in green cartoons.; **(B)** Toll like Receptor 5(TLR-5) immunological receptors used in this study. Respective Ramachandran plots also also shown with predicted structures **(C)** The docked complex of multi-epitope vaccine and TLR-5 is shown where the hotspot residues used (see Methods section for details) for data driven docking have been shown in spheres and colored in magenta and cyan for multi-epitope vaccine construct and TLR-5 receptor respectively.

The tertiary structure of the final multi-epitope vaccine construct and the immunogenic TLR-5 receptor was predicted using a deep learning approach of Alphafoldv2.0 program (42, 43). In order to confirm the quality of predicted structures, we calculated the Ramachandran plot of the modeled TLR-5 and Vaccine construct. For the multi-epitope vaccine construct, out of 309 residues, 85.1% fall in the core and favoured/acceptable regions, and 14.1% residues fall in additional allowed regions, and the remaining 0.8% residues fall under disallowed regions, see **Figure 2A**. However, for TLR-5 receptor, 81.2% of amino acids fall in the core acceptable region and the other 18.6% fall under the allowed region and with an additional 0.2% falling under the generously allowed region of the Ramachandran plot, see **Figure 2B**. Further, protein structure analysis (ProSA) webtool (58) was used in order to quantify the overall and local model quality of TLR-5 and multi-epitope vaccine construct, see **Figures S1**, **S2**.

The invocation of an appropriate immune response relies on the interaction between an antigenic molecule (in this case, designed multi-epitope vaccine) and a specific immune receptor (in this study, TLR-5 receptor). Molecular docking of the multi-epitope vaccine peptide with the modeled TLR-5 receptor was performed using the HADDOCK 2.2 web server (44) in order to evaluate the interaction between vaccine polypeptide and TLR-5 receptor and consequently the development of an immune response. The data-driven docking of designed multiepitope-TLR-5 complex were performed. The bacterial flagellin adjuvant is known to specifically bind to TLR-5 that stimulate and illicit response by innate immune system (56, 59). We have specifically used the information-driven docking where the knowledge about the specific residues involved in the interaction is needed to drive the docking calculations. In this regard, a recent study by Jacchieri and coworkers (59) employed a complementary hydropathy between the sequences of flagellin protein and TLR-5 receptor protein to predict the binding sites and structure of the complex. Further, their studies have clearly shown the potential binding sites in flagellin possessing “LQRVRELAVQ” sequence and for TLR5, a potential binding sites with “EILDISRNQL” sequence has been predicted. Further, their studies have shown that the side chains of Gln89, Arg92, and Glu93 on the surface of flagellin are optimally positioned to interact with the side chains of Glu552, Asp555, and Arg558 respectively on the surface of the predicted TLR-5 binding site.

The multi-epitope vaccine construct was docked with TLR-5 receptor using HADDOCK program which resulted in clustering 83 structures in 11 cluster(s), which represents 41% of the water refined HADDOCK generated models. We have specifically chosen the top ranked cluster with the lowest HADDOCK score which is most significant one for docking analysis. The HADDOCK score of − 73.4± 4.1 indicates a good interaction between the multi-epitope vaccine construct and TLR-5 receptor. The score of buried surface area (BSA) is 1784.5 ±  97.8 Å^2^ that reflected close proximity and a less water-exposed region on the protein binding surface. The **Figure 2C** show molecular docking between multi-epitope vaccine constuct and Human TLR-5 receptor. In order to study the stability of the docked complex we subjected the docked complex for Molecular Dynamic (MD) simulations, see below for detailed analysis.

### MD Simulations Predicts a Stable TLR-5 and Multi-Epitope Vaccine Interaction

**Figure 3:**
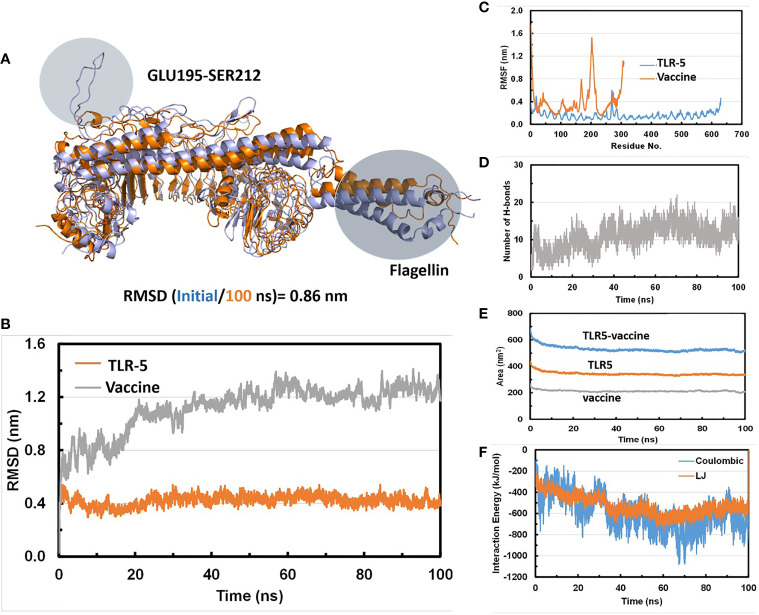
**(A)** Superimposition of a snapshot obtained after 10 ns and 100ns of simulation time highlighting a high RMSD value due to flexible loop regions and terminal region of flagellin in a vaccine construct; **(B)** Time evolution of backbone RMSD of TLR-5 and vaccine construct during MD simulations; **(C)** Backbone RMSF plots; **(D)** Time evolution of number of hydrogen bonds Between TLR-5 and vaccione construct; **(E)** Solvent accessible surface area of TLR-5 and vaccine construct; **(F)** Variation in Interaction energy plot with segregated electrostatics and vanderwaals component obtained for entire simulation time.

Molecular dynamics (MD) simulations have been proven to be very useful and effective method in order to study the stability and analysis of biological systems (60). In fact, our research group has successfully utilized and employed MD simulations to check the stability of different protein complexes (61–63) and nucleic acid systems (64–67). In this study, we used the GROMACS (45) software for molecular dynamics simulation to understand the structural properties and interaction between TLR-5 and the predicted multi-epitope vaccine. The simulation of TLR-5 immune receptor docked with multi-epitope vaccine was performed in order to analyze the interaction pattern of multi-epitope vaccine and changes that are induced in the complex after the initial docked conformation. In order to confirm the stability of TLR-5 immune receptor with the predicted multi-epitope vaccine, three MD simulations, each of 100 ns and starting with different velocities were conducted. Thus, in total, 300 ns of total simulation time was performed for TLR-5 complexes with multi-epitope vaccine construct. A 100 ns trajectory was used to analyze some essential parameters; the first one being the RMSD that reflect the stability between the two complexed moieties. The root mean square deviation (RMSD) values predict the fluctuation of Cα atoms of TLR-5 and Vaccine. In particular the RMSD value of the TLR-5 and vaccine construct complex after 10 ns and after 100ns simulations is 0.86 nm. The high RMSD can be correlated with the presence of loops region in the vaccine construct, see **Figure 3A**. The TLR-5 receptor, see **Figure 3B** shows the RMSD ranging from 0.33 nm to 0.54 nm, after 20 ns of time interval, which is considered as a mild fluctuation. In particular, the RMSD for the TLR-5 is 0.43 ± 0.03 nm. **Figure 3B** clearly indicates that TLR5 is stable throughout the simulation. However, an increased RMSD of 1.19 ± 0.08 nm was observed for multi-epitope vaccine construct. The high RMSD of multi-epitope vaccine could be correlated with the presence of loop regions where the epitope and linker sequences were inserted, that consequently resulted in the prominent fluctuations in vaccine. However, it should be noted that the RMSD of multi-epitope vaccine becomes stabilized after 20 ns of simulation time. The second analyzed parameter was Root Mean square fluctuation (RMSF) that represents amino acids side chains fluctuations. The RMSF parameter was plotted for TLR-5 and vaccine complex separately. Elevated fluctuations at the plot indicates highly flexible regions while mild ones correspond to regions that are more rigid. From **Figure 3C**, it seems evident that the calculated RMSF values for for Cα atoms of TLR-5 remained stable for the overall structure. In contrast, a high fluctuation was observed for residues ranging from 183 to 220 with an average RMSF of 0.74 ± 0.4 nm, which corresponds to the loop regions in multi-epitope vaccine structure. Next, we focused on the analysis of the number of hydrogen bonds between TLR5- and vaccine construct complex, which remained constant after 20 ns of the simulations, see **Figure 3D**. From this analysis, it can further be concluded that the after initial 20 ns was required in order to maintain a stable contact between TLR5 and vaccine construct. **Figure 3E**, evidenced that the large solvent accessible surface area was observed for the vaccine, which is followed by TLR-5. Intrigued by these observations, we calculated the Interaction energies between TLR5 and vaccine complex and further decomposed the interaction energy component into coulombic interactions [E(Coul)], which represents the interaction accounting for the electrostatics between TLR-5 and vaccine construct. The other energy component is Lennard Jones [E(LJ)] potential, that accounts for the Van der waals interactions between TLR-5 and vaccine complex. From **Figure 3F**, it seems evident that the predominant interaction that stabilizes the overall TLR-5 and vaccine complex stems from electrostatic component of the interaction, which seems to play a major role in binding. This energetic analysis was in line with the number of the hydrophilic-hydrophobic residues contacts that exists between the interface region of TLR-5 and multi-epitope vaccine construct.

### Detailed Analysis of Snapshots Obtained During MD Simulations and Its Comparison With Initial Modeled TLR5-Vaccine Construct

**Figure 4:**
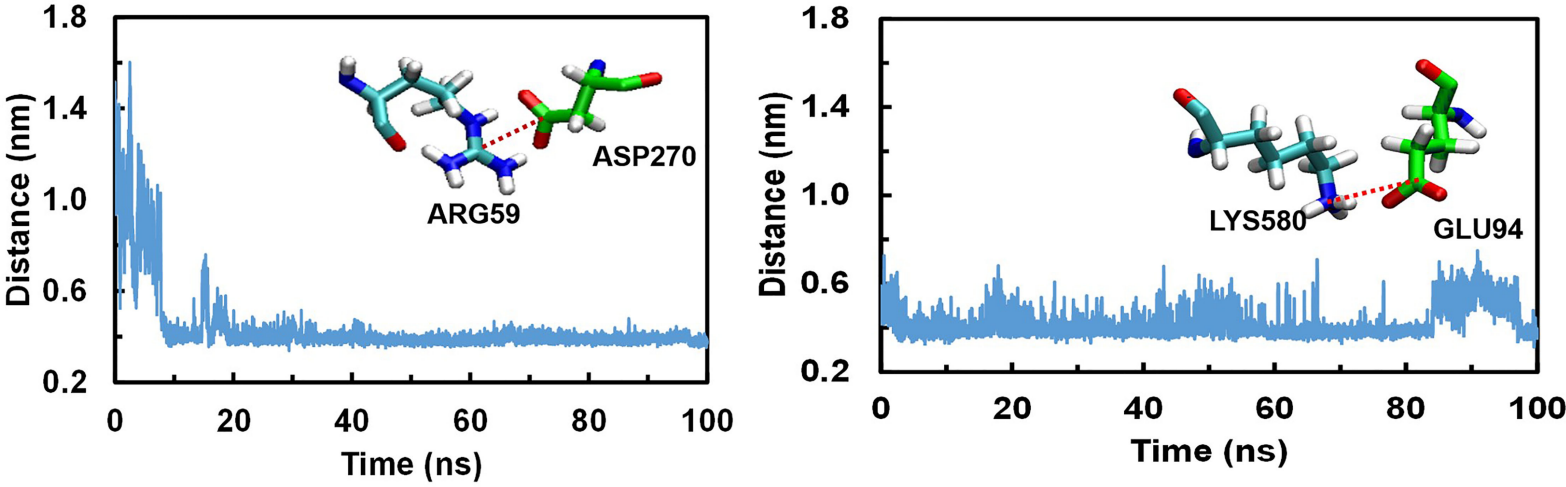
Conserved Salt bridge interaction contacts between TLR-5 and Vaccine construct. Left) Distances over time evolution between the Arg59(CZ)-Asp270(CB) atoms; Right) Distances over time evolution between Lys580(CE) and Glu94(CB) atoms.

**Figure 5:**
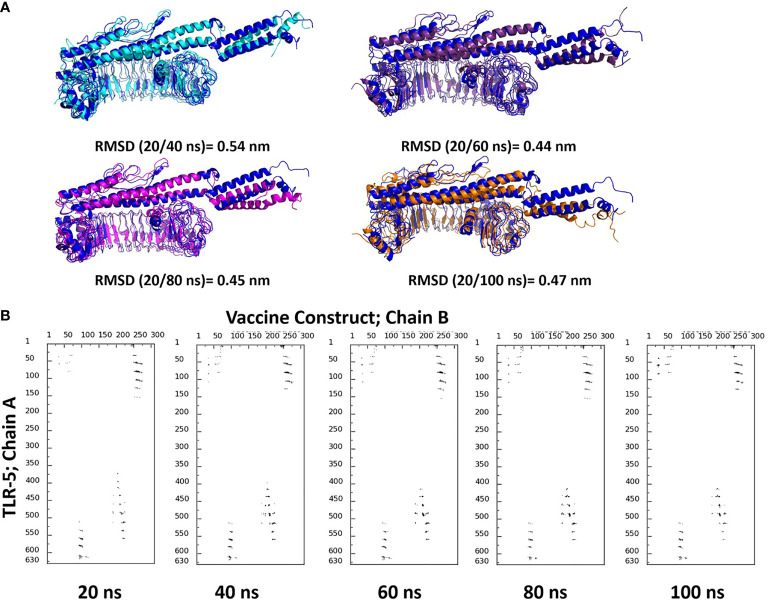
**(A)** Superimposition of selected snapshots of TLR-5 and vaccine construct and their respective RMSD values are also reported; **(B)** Contact maps showing the conservation of contacts between residues in between TLR-5 and Vaccine construct.

We analysed the possible non-covalent interactions between the multi-epitope vaccine and immune receptor TLR-5 using an in-house script. The docked complex is stabilized by a network of 12 hydrogen-bonding interactions, six hydrophobic interactions and five salt-bridge interactions within 5Å and 6Å distance cut-off respectively. The salt-bridge interaction exists between Arg59 of TLR-5 and ASP270 of Vaccine construct; His511 of TLR-5 and ASP227 of vaccine construct; ARG557 of TLR-5 and ASP227 of vaccine construct; LYS580 of TLR-5 and GLU94 of vaccine construct; ASP614 of TLR-5 and ARG91 of vaccine construct. In fact, the salt-bridge interaction between Arg59A of TLR-5 and ASP270B of Vaccine construct and LYS580A of TLR-5 and GLU94B of vaccine construct remained stable throughout the simulation time, see **Figure 4**. This analysis clearly revealed that the predicted vaccine shows a good interaction with TLR-5. To further assess the stability of the overall complex during the simulation time we computed RMSD of complex at different time steps and the comparative analysis of selected snapshots indicated the stability of overall TLR-5 and vaccine complex see **Figure 5A**. Moving forward, a detailed analysis of TLR-5 and vaccine docked construct interface was performed by the COCOMAPS tool (68, 69), see **Figure 5B**. COCOMAPS enables the analysis and visualization of the interface of interaction in protein complexes by making use of intermolecular contact maps in order to identify hot spot residues (68, 69). It seems evident by looking at **Figure 5B**, that overall contacts remained stable throughout the simulation time, and the analysis is shown for the selected snapshots, where the interaction pattern with the specific contacts remained stable, as shown by black patches in the graph. The interface area is 1261.65 Å^2^, with a percentage of polar and non-polar residues at the interface of approximately 64% and 36% respectively. Both the TLR-5 and vaccine construct interfaces involved in the interaction present a high percentage of hydrophilic residues. Using a cut-off distance of 8Å to define two atoms in contact, the dominant number of contacts exists between hydrophilic-hydrophobic residues that amounts to 230, which is followed by hydrophilic-hydrophilic and hydrophobic-hydrophobic contacts amounting to 222 and 52 respectively.

Finally, the similar analysis was performed for the docked complex by extracting a snapshot at 100ns of simulation. The TLR-5 and vaccine complex at the end of simulation was stabilized by a network of 32 hydrogen-bonding interactions, 12 hydrophobic interactions and 2 salt-bridge interactions within 5Å and 6Å distance cut-off respectively. The salt-bridge interaction exists between Arg59 of TLR-5 and ASP270 of multi-epitope vaccine construct, with an average distance between Arg59(CZ)-Asp270(CB) of 0.44 ± 0.2 nm. Next, a second salt bridge interaction between LYS580 of TLR-5 and GLU94 of vaccine construct with an average distance of Lys580(CE) and Glu94(CB) to be 0.42 ± 0.1 nm. In fact, both these salt-bridge interactions between TLR-5 and vaccine construct remained stable throughout the simulation time, see **Figure 4**. Further, a detailed analysis of TLR-5 and vaccine docked construct interface was performed by the COCOMAPS tool (68, 69). The interface area was increased from an initial 1261.65 Å^2^ (the initial modeled complex) to 1763.6 Å^2^. Interestingly, the percentage of polar and non-polar residues at the interface of approximately 62% and 38% respectively, which are nearly the same as of the initial model (64% and 36% respectively for polar and non-polar residues). Similar to the initial model, where employing a cut-off distance of 8Å to define two residues in contact, the dominant number of contacts still exists between hydrophilic-hydrophobic residues that amounts to 309, which was 230 in the initial modeled structure, which is followed by hydrophilic-hydrophilic and hydrophobic-hydrophobic contacts amounting to 222 and 52 respectively, and which were 260 and 85 in the initial modeled structure. This analysis clearly indicates that the overall interaction pattern of TLR-5 and multi-epitope vaccine construct remained stable throughout the simulation time.

### Peptide Synthesis and Conjugation

GenScript’s microwave-based PepPower™ technology was used to synthesize the selected peptode possessing the maximum sequence identity with all the considered DENV 1-4 serotyoes, see **Table 2**. The Cysteine was added to the C-terminus of immunogenic KREKKLGEFGKAKG epitope peptide. The peptide antigen with added Cysteine at the C-terminal is now CKREKKLGEFGKAKG was subsequently conjugated with KLH (Keyhole Limpet Hemocyanin) protein to immunize two rabbits at Genscript corporation (Piscataway, NJ, USA).

**Table 2:** Selected conserved epitope peptide for synthesis.

Epitope selected	Serotype	Protein name	Positions	IEDB sequence Identity
KREKKLGEFGKAKG	1	sp|P33478|POLG_DEN1S Genome polyprotein OS=Dengue virus type 1 (strain Singapore/S275/1990)	2947-2960	100
	2	sp|P07564|POLG_DEN2J Genome polyprotein OS=Dengue virus type 2 (strain Jamaica/1409/1983)	2947-2960	100
	3	sp|Q6YMS4|POLG_DEN3S Genome polyprotein OS=Dengue virus type 3 (strain Sri Lanka/1266/2000)	2946-2959	100
	4	sp|Q58HT7|POLG_DEN4P Genome polyprotein OS=Dengue virus type 4 (strain Philippines/H241/1956)	2944-2957	92.86

### *In-Vivo* Validation for Antibody Development and Assessment

**Figure 6:**
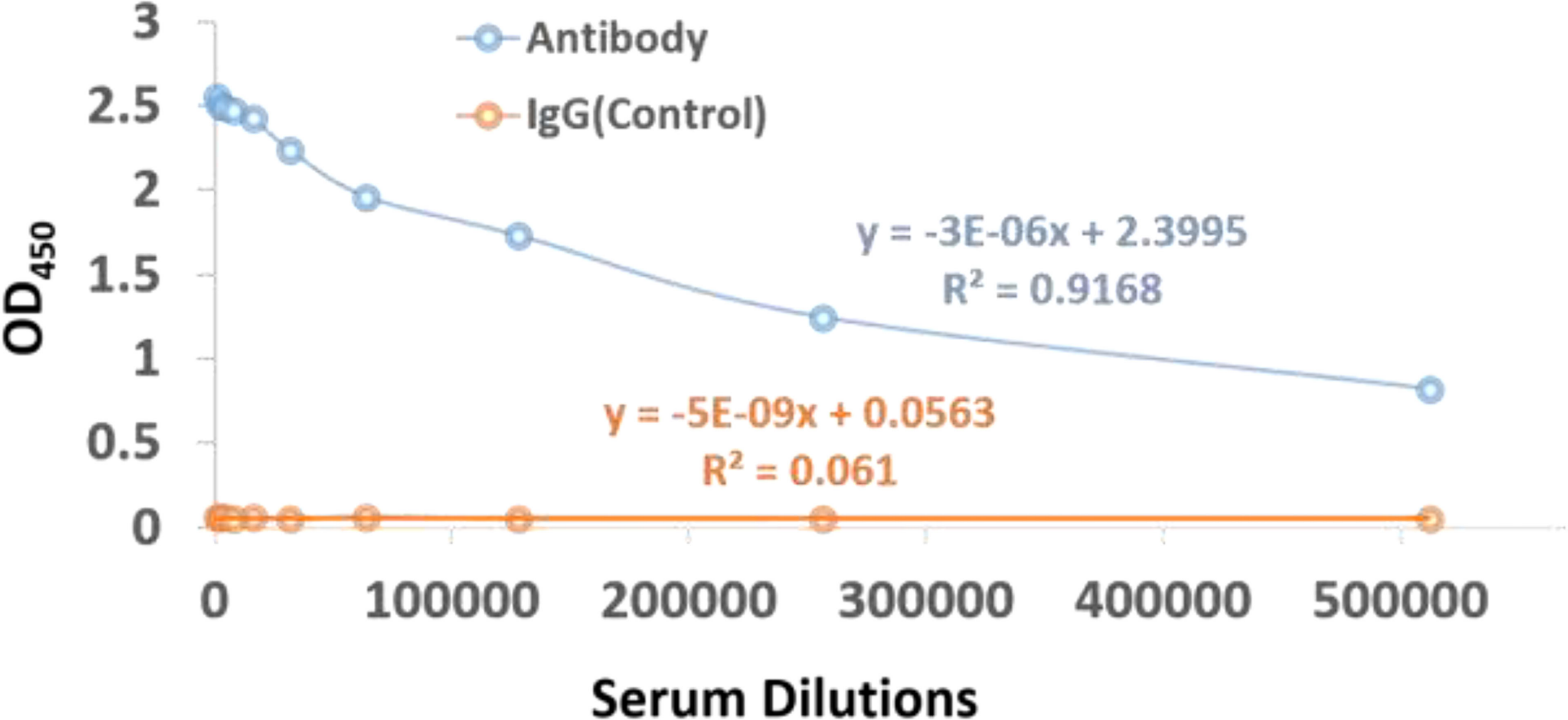
ELISA assay scatter plot of OD_450_ values and serum dilutions from (1:1000 to 1:512,000) of dengue virus antibody and IgG control with varying concentration of antigen. The OD_450_ values can be inversely correlated to the serum dilutions.

The affinity-purified concentration of the antibody is found to be 7.16mg in 4ml of the antiserum of subcutaneously immunized New Zealand white rabbits with KLH conjugated peptide. In order to confirm whether the KLH conjugate peptide can stimulate humoral immunity and produce antibodies, the Indirect ELISA was performed to determine the antibody titer. The antibody titre is expressed as the inverse of the highest dilution of the antibody which gives positive result in ELISA. The serum collected from rabbit is serially diluted more than 8 folds, which give positive results on ELISA. On the other hand, no color is observed when IgG in the range of 1:1,000 to 1:512,000 are incubated with the antigen, see **Figure 6**. These results clearly indicate that the imunogen designed in this study could stimulate the production of antibodies *in vivo*. Interestingly, the 10 fold diluted aliquot (i.e., 1: 520,000) is also observed to give positive result on ELISA, as indicated by the titer value of 14.67 with reference signal/blank >=2.1; (see **Supplementary Information File** for more details) which further suggest that the vaccine designed possesses good immunogenicity.

## Conclusion

In this study we have employed robust immunoinformatics approach coupled with *in vivo* studies in order to design a potential multi-epitope vaccine candidate for DENV vaccine. As a first step, we predicted 13501 MHC II binding CD4+ epitope peptides as a first step. Further we predicted and refined 10 conserved epitopes among all the four serotypes of the DENV and all the predicted epitopes are predicted to be interferon-inducing peptides. The antigenic and nontoxic epitopes ‘ATFTMRLLSPVRVPN’, ‘KREKKLGEFGKAKG’ and TFTMRLLSPVRVPNY that were conserved among all the four DENV serotypes were selected for final multi-epitope vaccine designing. The final vaccine has 309 amino residues, molecular weight 32.66 kDa, theoretical pI 9.99, and an estimated half-life of 30 hours in mammalian reticulocytes, and with a predicted antigenic score of 0.463. The molecular docking and molecular dynamic simulations predicts a stable complex between the multi-epitope vaccine construct and TLR-5 receptor. Overall, our computational analysis suggests that the constructed multi-epitope vaccine was highly immunogenic, safe, and non-toxic in nature. As a next step, the most conserved epitope KREKKLGEFGKAKG among the four DENV serotypes was synthesized. Finally, in order to confirm whether the KLH conjugate peptide can stimulate humoral immunity and produce antibodies, the Indirect ELISA was performed to determine the antibody titer. The affinity-purified concentration of the antibody is found to be 7.16mg in 4ml of the antiserum of subcutaneously immunized New Zealand white rabbits with KLH conjugated peptide. Interestingly, the 10 fold diluted aliquot (i.e., 1: 520,000) is also observed to give positive result on ELISA, as indicated by the titer value of 14.67. These results clearly indicate that the imunogen designed in this study could stimulate the production of antibodies *in vivo*.

## Data Availability Statement

The original contributions presented in the study are included in the article/**Supplementary Material**. Further inquiries can be directed to the corresponding authors.

## Ethics Statement

The animal study was reviewed and approved by Genscript corporation (Piscataway, NJ, USA).

## Author Contributions

MC and VK designed the whole project and analysis. LRG, SKG, ARS, UK, LC, MC carried out the analysis. SG, AS, MC wrote the manuscript. MC and ARS prepared figures. LC and MC supervised the overall study. All authors contributed to the article and approved the submitted version.

## Funding

This research received no external funding. The APC charges was funded by KAUST baseline research funding (to LC). The research reported in this publication was supported by funding from King Abdullah University of Science and Technology (KAUST). For computer time, this research used the resources of the Supercomputing Laboratory at King Abdullah University of Science & Technology (KAUST) in Thuwal, Saudi Arabia. Authors would also like to acknowledge team members from STEMskills Research and Education Lab Private Limited for critical reading of manuscript and computational support.

## Conflict of Interest

Authors UK, and ARS were employed by the company STEMskills Research and Education Lab Private Limited, Faridabad, Haryana, India.

The remaining authors declare that the research was conducted in the absence of any commercial or financial relationships that could be construed as a potential conflict of interest.

## Publisher’s Note

All claims expressed in this article are solely those of the authors and do not necessarily represent those of their affiliated organizations, or those of the publisher, the editors and the reviewers. Any product that may be evaluated in this article, or claim that may be made by its manufacturer, is not guaranteed or endorsed by the publisher.
