# Supplementary material for: Immunoinformatics Aided Design and In-Vivo Validation of a Cross-Reactive Peptide Based Multi-Epitope Vaccine Targeting Multiple Serotypes of Dengue Virus
Source: Front Immunol. 2022 Jun 21;13:865180. doi: 10.3389/fimmu.2022.865180 (PMC9254734; doi:10.3389/fimmu.2022.865180)
Supplement: Supplementary file 1 [file DataSheet_1.pdf]

Immunoinformatics Aided Design and In Vivo Validation of a Cross-Reactive Peptide Based Multi-Epitope Vaccine Targeting Multiple Serotypes of Dengue Virus

Vikas Kaushik[1], Sunil Krishnan G[1], Lovi Raj Gupta[1], Utkarsh Kalra[2, 3], Abdul Rajjak Shaikh[2]*, Luigi Cavallo[4]*, Mohit Chawla[4]*

[1]Domain of Bioinformatics, School of Bio-Engineering and Bio-Sciences, Lovely Professional University, Punjab, India 2) STEMskills Research and Education Lab Private Limited, Faridabad 121002, Haryana, India 3)Department of Data Science, Innopolis University, Republic of Tatarstan 420500, Russia, 4) King Abdullah University of Science and Technology (KAUST), Physical Sciences and Engineering Division, Kaust Catalysis Center, Thuwal 23955-6900, Saudi Arabia.

* Corresponding authors: razzaqsk@gmail.com(ARS); luigi.cavallo@kaust.edu.sa(LC); mohitchawla.bt@gmail.com(MC)

Table 1: Selected epitope peptide physio-chemical properties.

**Peptide Properties**	**Results**		
Sequence	KREKKLGEFGKAKG	ATFTMRLLSPVRVPN	TFTMRLLSPVRVPNY
Mass	1677.9320	1700.9367	1800.9269
Isoelectric point (pI)	10.43	12.48	11.48
Half-life(mammalian reticulocytes) hours	1.2	4.4	3.4
Grand average of hydropathicity (GRAVY)	-1.420	0.307	2.307
Aliphatic index	32.67	97.33	98.35
Net charge	+4	+2	+1
Hydrophobicity	+31.94 Kcal/mol	+8.34 Kcal/mol	+6.44 Kcal/mol
Hydropathicity	-1.7	0.31	0.25
The total number of negatively charged residues (Asp + Glu):	2	0	1

Total number of positively charged residues (Arg + Lys)	6	2	4

Different Non-covalent interactions identified in the docked complex stabilizing the TLR-5 (chain A) multi-epitope vaccine complex (Chain B); AA stands for Amino acids in respective chains.

Hydrophobic Interactions within 5 Å distance

Position	*AA*	*Chain*	*Position*	*AA*	*Chain*
35	PHE	A	251	ILE	B
57	TYR	A	258	VAL	B
81	TYR	A	258	VAL	B
487	LEU	A	217	PRO	B
561	LEU	A	100	ALA	B
561	LEU	A	97	VAL	B

H-bonding between TLR-5 and vaccine construct with respective donor-acceptor distances

**POS**	**CHAIN**	**AA**	**ATOM**	**POS**	**CHAIN**	**AA**	**ATOM**	**D-A**
559	A	GLN	NE2	219	B	TYR	O	3.18
559	A	GLN	NE2	219	B	TYR	O	3.18
98	B	GLN	NE2	611	A	VAL	O	3.39
98	B	GLN	NE2	611	A	VAL	O	3.39
259	B	ASN	ND2	81	A	TYR	O	2.93
259	B	ASN	ND2	81	A	TYR	O	2.93
59	A	ARG	NE	270	B	ASP	OD1	2.98
59	A	ARG	NE	270	B	ASP	OD2	3.19
59	A	ARG	NH2	270	B	ASP	OD1	2.63
59	A	ARG	NH2	270	B	ASP	OD1	2.63

580	A	LYS	NZ	94	B	GLU	OE2	2.81
609	A	TYR	OH	94	B	GLU	OE2	3.26

Ionic Interactions within 6 Å distance

Position	*AA*	*Chain*	*Position*	*AA*	*Chain*
59	ARG	A	270	ASP	B
511	HIS	A	227	ASP	B
557	ARG	A	227	ASP	B
580	LYS	A	94	GLU	B
614	ASP	A	91	ARG	B

Cation-Pi Interactions within 6 Å distance

Position	*AA*	*Chain*	*Position*	*AA*	*Chain*
219	TYR	B	537	ARG	A

Ionic Interactions within 6Å distance

Position	*AA*	*Chain*	*Position*	*AA*	*Chain*
59	ARG	A	270	ASP	B
511	HIS	A	227	ASP	B
557	ARG	A	227	ASP	B
580	LYS	A	94	GLU	B
614	ASP	A	91	ARG	B

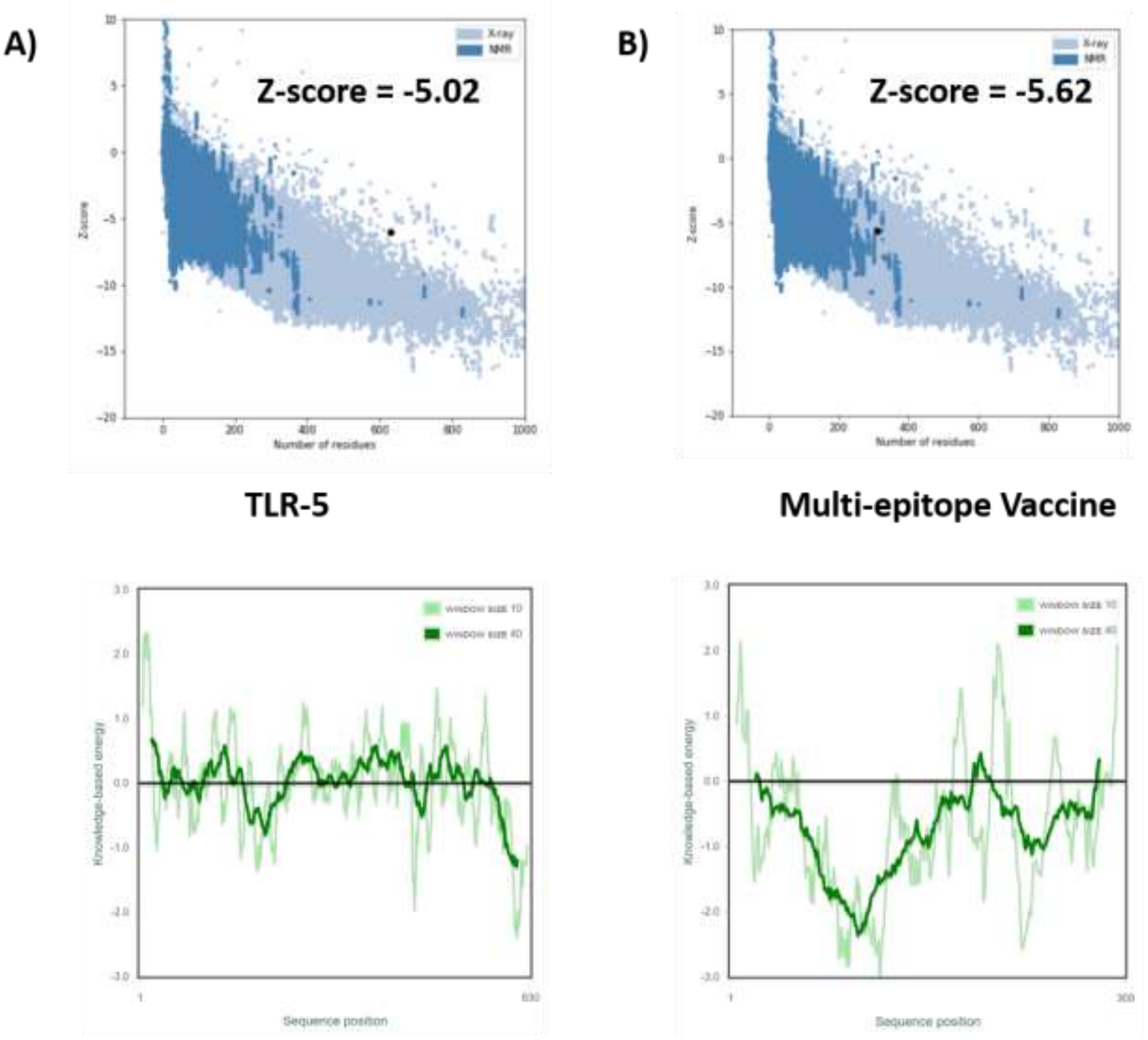

Figure S1: Global model quality and local model quality parameters predicted from Pro-SA web tool for the predicted Human TLR-5 and Multi-epitope vaccine structure.

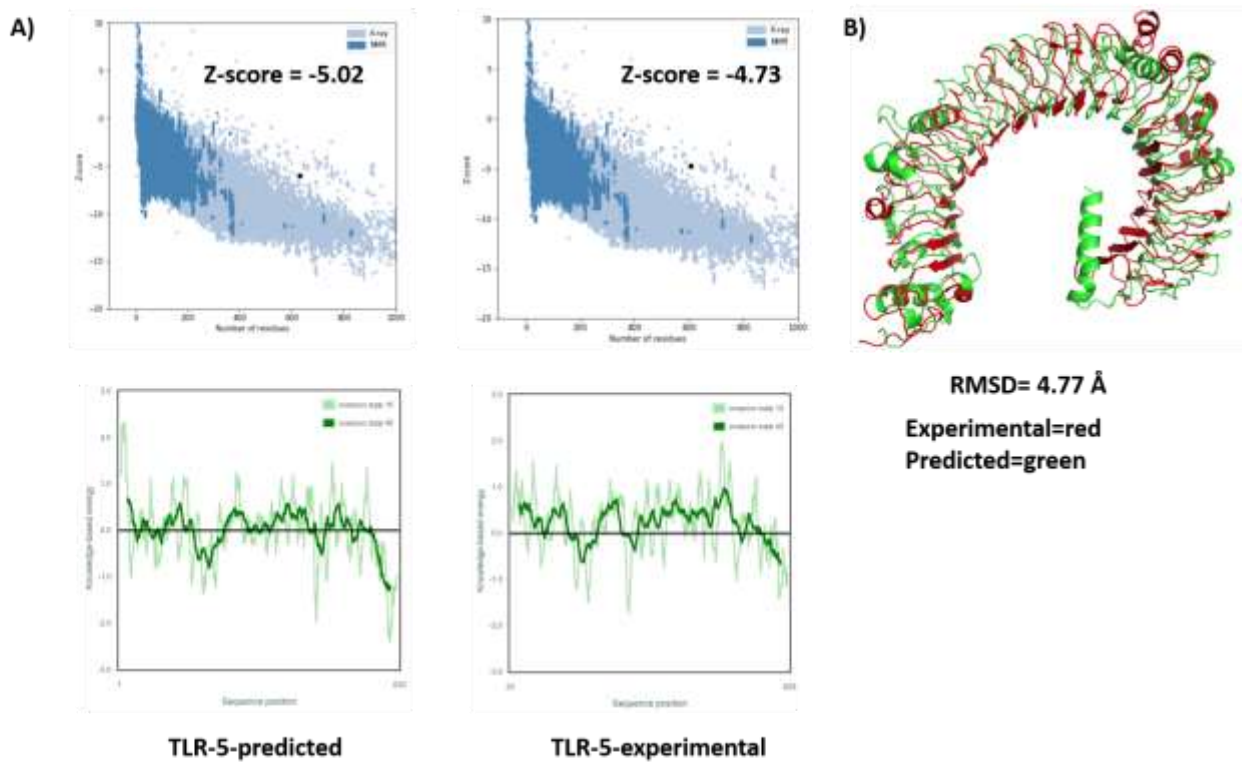

Figure S2: Global model quality and local model quality parameters predicted from Pro-SA web tool for the A) computationally predicted experimentally solved Human TLR-5 receptor; B) Superimposition of Computationally predicted (green) and experimentally solved (red) TLR-5 structure with corresponding RMSD value.

Certificate of Analysis

CONFIDENTIAL

SC1649-PU-Antibody Purification

Order ID: U964PGG210-7

Report From:

GenScript USA Inc

860 Centennial Ave

Piscataway, NJ 08854

United States of America

For research use only

CONFIDENTIAL

1. Project Information

Order ID: U964PGG210-7
Antigen Name: Antigen 2
Sequence: CKREKKLGEFGKAKG (Lot: U640SGG290-1)
Immunogen: Peptide-KLH conjugate
Host Strain: New Zealand Rabbit

2. Product Information

	Product Name	Detail	Form	Concentration (mg/ml)	Volume (ml)	Quantity (mg)	Purity	Aliquot	Preservative	Buffer
**Product 1**	/	Antigen (U640SGG290-1)	Lyophilized	/	/	2.00	/	1	/	/
**Product 2**	Rabbit antibody	Rabbit IgG control (Whole Molecule)	Lyophilized	/	/	0.10	/	1	0.02% Sodium Azide	PBS
**Product 3**	Rabbit antibody	Affinity-Purified antibody	Liquid	1.791	4.00	7.16	98%	1	0.02% Sodium Azide	PBS

Buffer PBS is short for Phosphate Buffered Saline (PBS, pH 7.4)
Concentration is measured by NanoDrop Spectrophotometer A280nm
Purity is measured by SDS-PAGE
Concentration, volume and quantity are all single vial data.

3. QC Results

Indirect ELISA
Coating Antigen(s): Free Peptide
Coating Concentration: 4ug/ml, 100 µl/well

CONFIDENTIAL

Coating Buffer: Phosphate Buffered Saline, pH 7.4

Secondary Antibody: Anti-Rabbit IgG Fc Monoclonal Secondary Antibody (Min X Hu, Ms, Rt, Sh, Bv, Gt, Camel) (HRP conjugate) (GenScript, Cat. No. A01856)

Table 1. ELISA results of Rabbit IgG control and purified antibody

Concentration (ng/ml)	1,000.00	500.00	250.00	125.00	62.50	31.25	15.62	7.81	3.90	1.95	Blank	/
**Dilution**	**1:1,000**	**1:2,000**	**1:4,000**	**1:8,000**	**1:16,000**	**1:32,000**	**1:64,000**	**1:128,000**	**1:256,000**	**1:512,000**	**Blank**	**Titer**
**Antibody**	2.550	2.496	2.489	2.470	2.424	2.234	1.961	1.731	1.247	0.822	0.056	>1:512000
**IgG**	0.058	0.057	0.057	0.052	0.058	0.051	0.063	0.054	0.054	0.054	0.058	<1:1000

The titer is the highest dilution with S/B (Signal/Blank) >=2.1, the OD450 in blank is the average of two technical replicates.
The starting concentration of 1 mg/ml and the corresponding dilution ratio is calculated based on the actual concentration.

4. Storage Condition

The antibody is stable at 2-8°C for up to 1 month. For long term storage, aliquot the antibody and store at -20°C or below. Avoid repeated freeze-thaw cycles. Antibody is stable for up to two years.

Date:09/03/2021

Approved by:

CONFIDENTIAL

Liping Ma

Quality Control Group, Antibody Department

CONFIDENTIAL
